# Icosapent Ethyl – A Successful Treatment for Symptomatic COVID-19 Infection

**DOI:** 10.7759/cureus.10211

**Published:** 2020-09-02

**Authors:** Amnon A Berger, Robert Sherburne, Ivan Urits, Haresh Patel, Jonathan Eskander

**Affiliations:** 1 Department of Anesthesia, Critical Care and Pain Medicine, Beth Israel Deaconess Medical Center, Harvard Medical School, Boston, USA; 2 Department of Anesthesia, Critical Care, and Pain Medicine, Beth Israel Deaconess Medical Center, Harvard Medical School, Boston, USA; 3 Critical Care Medicine, Maryview Medical Center, Portsmouth, USA; 4 Anesthesiology and Pain Medicine, Portsmouth Anesthesia Associates, Portsmouth, USA

**Keywords:** covid-19, inflammation, ards (acute respiratory distress syndrome), omega-3, hypertriglyceridemia, cytokine release syndrome (crs)

## Abstract

COVID-19 is a fatal, universal pandemic caused by the SARS-CoV-2 virus that has directly caused at least 95,235 deaths in the US by May 2020. It has a poor prognosis with a mortality rate as high as 21% in the general population at the height of the pandemic, a rate that is much higher in elderly patients, as well as those requiring intensive care unit (ICU) care. The role of inflammation in symptomatic COVID-19 is being studied, and it is hypothesized that hyper-inflammation is a causative factor in severe COVID-19 disease. Treatment options are limited and mostly rely on supportive care.

Icosapent ethyl (IPE) is an Omega-3 fatty acid derivative that has been shown to significantly reduce cardiovascular mortality and is used as an adjunct to statin therapy. Though it has been shown to act as an anti-inflammatory, it is not currently indicated for that purpose. Here, we describe, for the first time, the successful treatment of a COVID-19 patient with IPE.

## Introduction

COVID-19 is a fatal, universal pandemic. Originating from Wuhan, the capital city of Hubei province in China, it is caused by the SARS-CoV-2 virus (previously 2019-nCov), which is efficiently transmitted from person to person. It most commonly causes a respiratory illness that could potentially deteriorate to adult respiratory distress syndrome (ARDS) [1-2]. The overall mortality from COVID-19 is reported to be anywhere between 2.1% and 21% [2-5], with specific vulnerability in patients above 70 years of age [3]. In the US, it is estimated to have directly caused 95,235 (of 122,300 excess) deaths from March to May 2020 [6]. In patients requiring intensive care unit (ICU) care, intubation, and ventilation, the prognosis is even poorer and only a small fraction survive [3,5,7].

Inflammation is hypothesized to play a causative role in symptomatic COVID-19 and evidence points to hyper-inflammatory states in patients [5]. This is further supported by preliminary results from the Randomized Evaluation of COVID-19 Therapy (RECOVERY; NCT04381936) trial [8] and the incorporation of dexamethasone in National Institutes of Health (NIH) treatment guidelines [9]. Unfortunately, outside of dexamethasone, treatment options for COVID-19 are severely limited. The anti-malarial drugs - chloroquine and hydroxychloroquine - were thought to provide some protection against viral spread. However, recent evidence suggests they may not confer the defense they were previously thought to [4]. Treatment with tocilizumab was also studied, but the literature on IL-6 inhibitors remains inconclusive [10-11]. The main treatment for COVID-19 remains mostly supportive.

Icosapent ethyl (IPE), a form of eicosapentaenoic acid (EPA), is indicated for the treatment of persistent hypertriglyceridemia, proven to reduce cardiovascular risk, and have been reported to have anti-inflammatory activity [12]. It is currently indicated as an adjunct to maximally tolerated statin therapy [13-15]. Since the triglyceride-lowering effect of IPE was only modest, the mechanism of cardiovascular risk-reduction is still unclear. One suggested mechanism is IPE's anti-inflammatory effect [16]. This is supported by results from the Evaluation of the Effect of Two Doses of AMR101 (Ethyl Icosapentate) on Fasting Serum Triglyceride Levels in Patients With Persistent High Triglyceride Levels (≥ 200 mg/dL and < 500 mg/dL) Despite Statin Therapy (ANCHOR; NCT01047501) and the Multi-Center, PlAcebo-Controlled, Randomized, Double-BlINd, 12-week study with an open-label Extension (MARINE; NCT01047683) trials, showing a reduction in inflammatory markers in patients under IPE treatment [17-19]. IPE is not currently indicated for anti-inflammatory treatment. Here, we report, for the first time, the use of IPE to treat a patient with COVID-19.

## Case presentation

A 53-year-old woman (Patient #1) with hyperlipidemia and no other known medical conditions presented to the clinic after confirmed exposure as well as a positive test for COVID-19. Her 21-year-old healthy daughter (Patient #2) was similarly exposed at the same time. Both women were of good health, had no other known risk factors for COVID-19, and had a body mass index (BMI) of 21 and 17, respectively. They had not shown any symptoms and received no treatment until the fourth post-exposure day. Four days after being exposed to a confirmed COVID-19 case, both developed persistent fevers up to 101F and later sore throat, nasal congestion, and cough on symptom day (SD) 2 and anosmia on SD4. On SD2, after obtainment of informed consent, Patient #1 began a course of oral IPE (2g twice daily). This treatment was offered to her following our success with this regimen in patients with inflammatory response and shock (report under review for publication). Her daughter (Patient #2) declined IPE. They otherwise received only symptomatic care that did not differ between the two.

**Figure 1:**
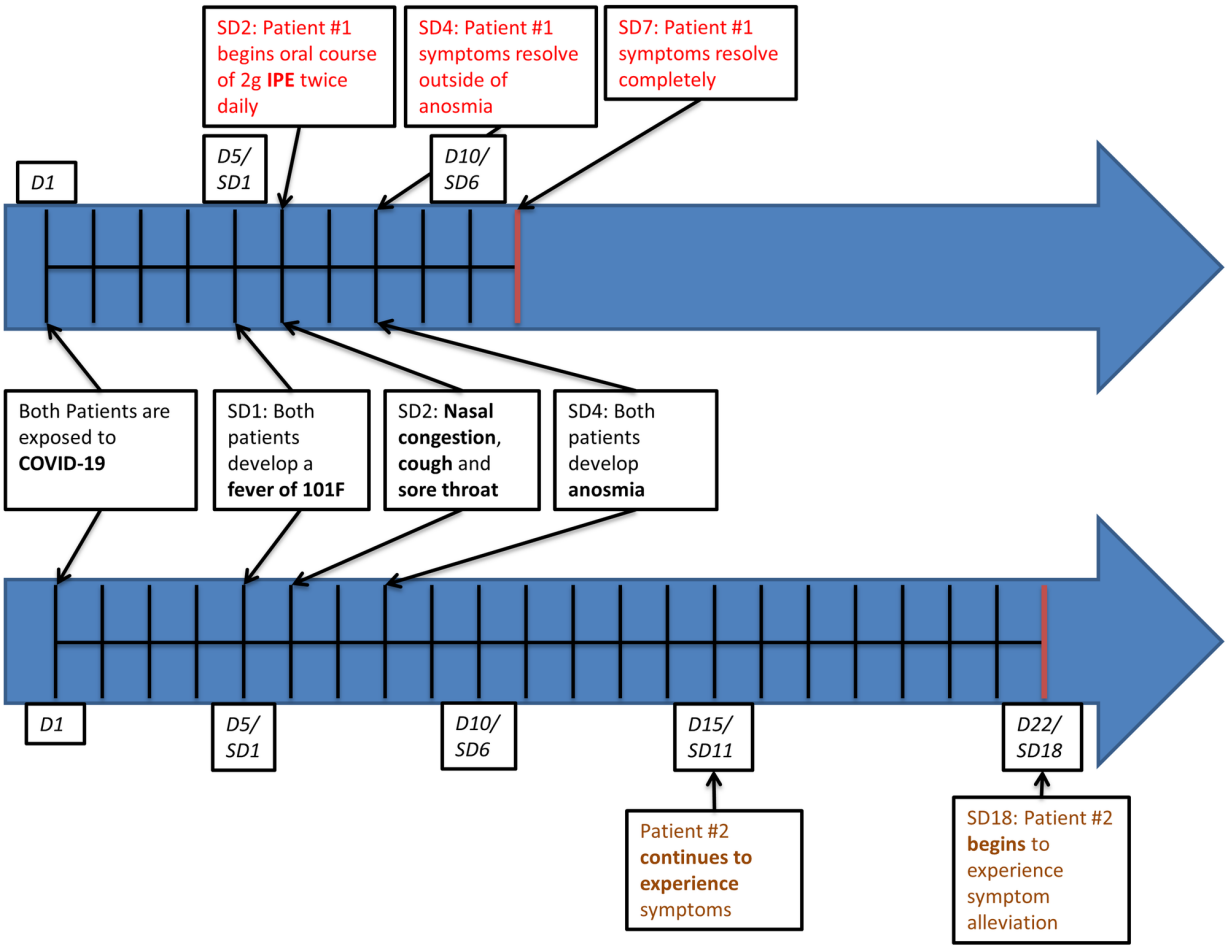
Timeline of symptoms development and alleviation The timeline summarizes the course of disease in both of our patients. Patient #1 elected to begin IPE therapy; her daughter (Patient #2) opted out of IPE therapy. Within five days of IPE therapy initiation, Patient #1 became and remained symptom-free from COVID-19. Her daughter continued to experience symptoms that only began to subside on SD18. D, Day; SD, Symptom Day; IPE, Icosapent Ethyl

Fevers began to subside on SD2, however, symptoms persisted; on SD4, the 53-year-old patient no longer reported any symptoms outside of anosmia, which has later resolved on SD7, five days after beginning treatment with IPE. In comparison, her 21-year-old daughter continued to experience sore throat, nasal congestion, and anosmia through SD18. She had only started experiencing partial relief on SD18, significantly after the complete resolution of symptoms in her mother (see red vertical lines in Figure 1). Figure 1 displays the course of disease in both patients and emphasizes the difference in disease development with and without IPE treatment.

## Discussion

These are two patients with similar genetic and medical backgrounds who differ in age only and neither would be considered elderly. They were exposed in the same manner to COVID-19, developed similar symptoms, but only one was treated with IPE and experienced much shorter symptoms duration. Surprisingly, and in contrast to our knowledge of COVID-19 so far, the older patient actually fared better. These moderate COVID-19 cases join our previous experience with severe, hypoxic COVID-19 and septic patients (case series of three other severe COVID-19 patients not yet published, case report for IPE use in severe pancreatitis currently under review), demonstrating the utility of IPE in acute inflammatory states and specifically with COVID-19 infections.

In the IPE Reduction of Cardiovascular Events with EPA-Intervention (REDUCE-IT; NCT1492361) trial designed to determine cardiovascular disease risk reduction, triglyceride-lowering was modest, thereby suggesting IPE mechanisms include an anti-inflammatory effect. This is supported by evidence of a significant reduction in inflammatory markers with the use of IPE in both the ANCHOR and MARINE trials, which were designed to evaluate the triglyceride-lowering activity of IPE, as well as previous evidence from EPA trials [12]. Immune modulation is the most likely action of IPE in our patients, however, IPE and EPA were previously also reported to improve endothelial function and have direct virucidal activity [20]. Though Patient #1 did have a medical history of hyperlipidemia, anti-lipid effects are less likely to be demonstrated in such a short timeline. Further research in larger groups of patients will likely contribute further to our understanding of the underlying mechanism of action.

## Conclusions

Though anecdotal, we report the first evidence of using IPE as a treatment for the symptoms associated with a COVID-19 infection. Further research is required to further explore the efficacy of IPE as a treatment for COVID-19. Our report is intended to report the successful treatment of COVID-19 with IPE in a single case in hopes to ignite such research.
